# Mucosal Immunization with Live Attenuated *Francisella novicida* U112Δ*iglB* Protects against Pulmonary *F. tularensis* SCHU S4 in the Fischer 344 Rat Model

**DOI:** 10.1371/journal.pone.0047639

**Published:** 2012-10-30

**Authors:** Aimee L. Signarovitz, Heather J. Ray, Jieh-Juen Yu, M. N. Guentzel, James P. Chambers, Karl E. Klose, Bernard P. Arulanandam

**Affiliations:** 1 South Texas Center for Emerging Infectious Disease and Center of Excellence in Infection Genomics, University of Texas at San Antonio, San Antonio, Texas, United States of America; 2 Department of Microbiology and Immunology, University of Texas Health Science Center at San Antonio, San Antonio, Texas, United States of America; Louisiana State University Health Sciences Center, United States of America

## Abstract

The need for an efficacious vaccine against *Francisella tularensis* is a consequence of its low infectious dose and high mortality rate if left untreated. This study sought to characterize a live attenuated subspecies *novicida*-based vaccine strain (U112Δ*iglB*) in an established second rodent model of pulmonary tularemia, namely the Fischer 344 rat using two distinct routes of vaccination (intratracheal [i.t.] and oral). Attenuation was verified by comparing replication of U112Δ*iglB* with wild type parental strain U112 in F344 primary alveolar macrophages. U112Δ*iglB* exhibited an LD_50_>10^7^ CFU compared to the wild type (LD_50_ = 5×10^6^ CFU i.t.). Immunization with 10^7^ CFU U112Δ*iglB* by i.t. and oral routes induced antigen-specific IFN-γ and potent humoral responses both systemically (IgG2a>IgG1 in serum) and at the site of mucosal vaccination (respiratory/intestinal compartment). Importantly, vaccination with U112Δ*iglB* by either i.t. or oral routes provided equivalent levels of protection (50% survival) in F344 rats against a subsequent pulmonary challenge with ∼25 LD_50_ (1.25×10^4^ CFU) of the highly human virulent strain SCHU S4. Collectively, these results provide further evidence on the utility of a mucosal vaccination platform with a defined subsp. *novicida* U112Δ*iglB* vaccine strain in conferring protective immunity against pulmonary tularemia.

## Introduction

Animal models for vaccine development should *1)* optimally reflect human susceptibility to the agent of interest and *2)* provide similar host responses to humans. In the case of the Gram negative pathogen *Francisella tularensis*, the vast majority of work has been conducted in mice [1]–[9]; however, mice are highly susceptible to all subspecies of *F. tularensis* including the human avirulent subspecies *novicida* (mouse LD_50_<10 CFU) and the highly human virulent subspecies *tularensis* strain SCHU S4 (mouse LD_50_<10 CFU). Thus, vaccine studies performed in the mouse model are often restricted to a narrow range of challenge inocula with SCHU S4. Previous studies have determined that “white rats” may serve as a more relevant platform for vaccine studies, as these animals were found to be more resistant to *F. tularensis* than mice, guinea pigs, and rabbits [10], [11]. Recently, we [12] and others [13], [14] have begun to characterize the Fischer 344 (F344) rat as a potential second rodent model for pulmonary tularemia vaccine studies. We have demonstrated that *F. tularensis* replicates within hepatocytes and bone marrow derived macrophages and that the rat may better reflect human susceptibility to pulmonary tularemia as evidenced by the LD_50_ of each subspecies when administered intratracheally (i.t.) [12]. Rats challenged i.t. with human virulent subspecies *holarctica* and *tularensis* strains exhibited a mean time to death of 10 days and pulmonary LD_50_ values of approximately 10^5^ and 500 CFU, respectively [12], as compared to the mouse where the LD_50_ of both subspecies is less than 10 CFU [4]. Moreover, rats exhibit similar susceptibility as humans to the other subspecies of *F. tularensis* (i.e., resistance to *F. novicida* and LVS) in comparison to mice [12], [13]. Thus, F344 rats may serve as a more reflective platform for evaluation of putative tularemia vaccine candidates.

To this end, a successful vaccine against a respiratory pathogen such as *F. tularensis* will require induction of protective mucosal immunity at the site of infection. Various routes have been exploited to determine the most effective site to stimulate mucosal and systemic immunity including ocular [15], sublingual [16]–[20], intranasal/intratracheal [5], [21]–[24], oral [25]–[29], intravaginal [30] and intrarectal [31]. For *F. tularensis*, the main routes of vaccination exploited have been subcutaneous [13], [32], intranasal/intratracheal [2], [3], [33], intradermal [34]–[37], and oral [27], [28], [37]. Protection against pulmonary *F. tularensis* challenge in animal models has demonstrated a role for both cellular and humoral arms of the immune system [38] since B cells [1], IgA and CD4^+^ T cells [27], [39], NK and CD8^+^ T cells [40] as well as IFN-γ [39], [41] and Th1 type responses have been shown by different investigators to assist in clearance of *F. tularensis*. To this end, protection also can be enhanced by the use of IL-12 as an adjuvant [42]. Among these vaccination routes, the oral and intranasal routes have received considerable attention for the ability to target microfold cells (M-cells), located in Peyer's patches within the gastrointestinal tract or nasal-associated lymphoid tissue (NALT) in the respiratory tract, as induction sites [43].

Given the establishment of the F344 rat model in our laboratory, we describe in this study the efficacy of *F. tularensis* subsp. *novicida* U112Δ*iglB* as a putative vaccine candidate by comparing two mucosal routes of vaccination (oral *vs.* i.t.). U112Δ*iglB* was generated using a targeted mutagenesis approach [44]. This mutant lacks the gene iglB, which is located within the iglABCD operon of the *Francisella* pathogenicity island (FPI). The FPI is comprised of 17 genes, found in duplicate copies in the highly human virulent subsp. *tularensis* and *holarctica*, and a single copy in subsp. *novicida* [45]. Genes within the pathogenicity island are required for intramacrophage replication, phagosomal escape, and virulence. The FPI gene iglB has been demonstrated to be part of a putative type VI secretion system in *F. tularensis* [46], [47]. Along with iglA, iglB forms an iglAB outer tubular structure which contracts around an iglC inner tubular structure through which secreted proteins are propelled into the host cell [47]. U112Δ*iglB* is attenuated both *in vitro* in J774 macrophages and *in vivo* with a LD_50_>10^7^ CFU in BALB/c mice (as compared to its parental strain U112, LD_50_<10 CFU [48]).

In this study, we sought to characterize the immune responses generated from mucosal vaccination (i.t. or oral) with either live U112 or the defined attenuated mutant U112Δ*iglB*. Oral vaccination of F344 rats with either U112 or U112Δ*iglB* generated 50% protection against subsequent pulmonary SCHU S4 challenge. In contrast, i.t. vaccination with U112 was 100% protective while i.t. U112Δ*iglB* was 50% protective against pulmonary SCHU S4 challenge. Overall, these findings reaffirm previous studies suggesting that mucosal routes of immunization may be efficacious means to provide protection against pulmonary tularemia.

## Materials and Methods

### Ethics Statement

All animal experiments were performed in compliance with the Animal Welfare Act, the U.S. Public Health Service Policy on Humane Care and Use of Laboratory Animals and “Guide for the Care and Use of Laboratory Animals” published by the National Research Council. All animal work was done in accordance with the guidelines set forth by the University of Texas at San Antonio Institutional Animal Care and Use Committee (IACUC) and Institutional Biosafety Committee (IBC), who specifically approved this study under approved protocol MU031-11/11A0.

### Animals

Six-week old female Fischer 344 rats were obtained from the National Cancer Institute (Frederick, MD). Animals were housed in ventilated cages in the University of Texas at San Antonio animal vivarium and received food and water *ad libitum*.

### Bacteria

*Francisella tularensis* subspecies *novicida* strain U112 was obtained from Dr. Francis Nano, University of Victoria, Canada. *F. tularensis* subspecies *tularensis* strain SCHU S4 was obtained from the Centers of Disease Control and Prevention, Atlanta, GA. The vaccine strain U112Δ*iglB* was identical to that previously described [44], [48]. All strains were grown from original stocks in tryptic soy broth (TSB) or tryptic soy agar (TSA) (both obtained from BD Biosciences) supplemented with 0.1% (w/v) L-cysteine (Fisher Scientific). Dilution plating was carried out on this medium to determine titers of bacterial stocks.

### Generation of primary rat macrophages

Bone marrow derived macrophages (BMDM) were obtained from four to six week old Fischer 344 rats (n = 3) sacrificed by CO_2_ asphyxiation followed by cervical dislocation. Femurs were collected aseptically and washed in Dulbecco's Modified Eagles Medium (DMEM; Mediatech, Fairfax, VA) containing 10% (w/v) fetal bovine serum (FBS; HyClone, Logan, UT) supplemented with L-glutamine (2 mM), (all contents together called D10) and penicillin/streptomycin (p/s; 100 U/mL and 100 µg/mL, respectively). The end of the femur was removed and pelleted marrow was flushed from the bone with 10 mL D10 plus p/s and then centrifuged. Supernatants were discarded and the marrow was washed twice with D10, resuspended in conditioned medium [DMEM containing 20% (w/v) FBS, L-glutamine, penicillin/streptomycin and 10% (w/v) supernatant from the L929 hybridoma cell line [49], placed in a 75 cm^2^ tissue culture flask, and incubated at 37°C in 5% CO_2_. After 24 hr, media containing non-adherent cells was decanted and transferred to a second flask to recover additional cells. The medium was replaced every two days until differentiation occurred, with the last two medium changes using conditioned medium without p/s. Cells were utilized 8 days after collection. Alveolar macrophages (ALVM) were isolated using a modification of the method of Engwall *et. al.* [50]. Briefly, rats (n = 3) were sacrificed as previously described and the dorsal aorta was severed distal to the kidneys. The chest cavity was opened to expose the lungs, and the trachea severed just below the larynx. The lungs and heart were removed and placed in 250 mL ice cold sterile PBS containing p/s for one hour. An 18-gauge 1½” plastic catheter sheath was carefully inserted in the trachea and tied in place using surgical thread. A 10 mL luer-lock syringe filled with 8 mL PBS containing p/s was attached to the catheter and the contents of the syringe gently injected into the lungs. Using the thumb and forefinger, the lungs were gently massaged, and then the injected solution was removed from the lungs using the syringe and the contents placed in a 50 mL conical tube on ice. This procedure was serially repeated using the total of 50 mL and the recovered lavage fluid was centrifuged to pellet cells. The supernatant was discarded and the cells were washed twice in 30 mL PBS with p/s, and then resuspended in 12 mL D10 with p/s, seeded into a 75 cm^2^ tissue culture flask, and incubated at 37°C. The medium was replaced after 24 hr, and the cells were maintained at 37°C for up to one week with media changes every 48 hr. Macrophages were collected by gentle scraping and subsequently stained to confirm differentiation with mouse anti-rat CD11b (OX-42) conjugated with AlexaFluor 647 (AF 647, AbD Serotec, Raleigh, NC) [51], mouse anti-rat CD11b (WT.5) conjugated with fluorescein isothiocyanate (FITC, BD Biosciences, San Diego, CA) [52], [53], or mouse anti-rat CD172 conjugated with phycoerythrin (PE; also designated as OX41; BD Biosciences) [51].

### Fluorescent bead assay

Rat BMDM and ALVM in D10 were seeded into 6 well plates (1×10^6^ cells/well) and allowed to adhere. Yellow-green (FITC) fluorescent (505/515 wavelength) carboxylate-modified microspheres (Invitrogen, Carlsbad, CA), 1.0 µm in diameter, were added to confluent cell monolayers at either 10 or 100 beads/cell and incubated for 2 hr to allow for phagocytosis. Extracellular beads were washed off, cells were collected by gentle scraping, and stained with either mouse anti-rat CD11b (OX-42)-AF 647 [BMDM] or mouse anti-rat CD172-PE [ALVM], and analyzed by flow cytometry [51].

### Phagocytosis assay

ALVM were suspended in D10, seeded into 96-well plates (2×10^5^ cells/well) and allowed to adhere. Confluent macrophage monolayers were infected with either WT U112 or the mutant U112*ΔiglB* strain (10 MOI) for 2 hr and washed twice with D10 prior to 1 hr of gentamicin treatment (20 µg/mL). Cells were washed three times with D10 and then incubated in D10 for up to 48 hr, a maximal time frame for replication of the bacteria. At indicated time points (3, 24, 48 hr post challenge), media was decanted and cells lysed with 0.2% deoxycholate solution. Intracellular bacteria were enumerated by plating serial dilutions of lysates on TSA containing cysteine.

### Vaccination and Challenge

Eight to nine week old Fischer 344 rats were anesthetized with 5% (v/v) isoflurane and oxygen at 2 liters per minute using a rodent anesthesia chamber (Harvard Apparatus, Hollister, MA). Rats were placed dorsally on a surgical platform (Alpha Lab Supply, Alberquerque, NM), and a laryngoscope (Penn Century, Inc., Philadelphia, PA) was inserted to assist in securing the tongue and allowing visualization of the trachea and esophagus. Intratracheal vaccination was utilized in this study to ensure inocula reached the lungs and initiated the infectious process. In contrast to humans, inhalation by the rat does not allow for as effective lower respiratory tract infection, as the turbinates of rat nasal passages increase nasal deposition [54]). Intratracheal vaccination/challenge was achieved using a 20-gauge plastic catheter attached to a blunt-ended needle inserted into the trachea allowing delivery of 100 µL inoculum followed by approximately 300 µL air to ensure the bacteria reached the lungs. Oral vaccination was achieved with the catheter inserted into the esophagus with delivery of 300 µL inoculum followed by approximately 300 µL of air to ensure that bacteria reached the stomach. Animals were allowed to awaken, returned to their cages, and monitored daily for morbidity and mortality following vaccination and challenge. Vaccination doses (10^5^ CFU for U112 and 10^7^ CFU for U112Δ*iglB* or LVS) and the challenge doses (approximately 1×10^4^ CFU SCHU S4) were similar for i.t. and oral routes of administration. Doses were chosen based on LD_50_ analyses following F344 pulmonary infection (SCHU S4 LD_50_ = 500 CFU and U112 LD_50_ = 5×10^6^ CFU [12], and U112Δ*iglB* LD_50_>10^7^ CFU, unpublished data). All vaccination and challenge inocula were dilution plated on TSA containing cysteine to verify doses.

### Bacterial Dissemination of the Vaccine Strain

Eight to nine week old Fischer 344 rats were i.t. or orally vaccinated as described with either 10^5^ CFU U112 or 10^7^ CFU U112Δ*iglB*. At days 3, 14, and 21 following vaccination, rats (n = 3 per group for each time point) were sacrificed, and lungs, livers, and spleens were collected post-mortem. Tissues were homogenized using a tissue homogenizer (Fisher Scientific) and dilution plated on TSA plus cysteine to enumerate organ bacterial burdens.

### Measurement of Antigen-Specific Cellular Responses

Eight to nine week old Fischer 344 rats (n = 3 per group) were vaccinated, rested, and monitored for 14 or 28 days. At those times, animals were euthanized and tissues (spleens, cervical lymph nodes [CLN], and mesenteric lymph nodes [MLN]) were collected from each group of animals and single cell suspensions of splenocytes and lymphocytes were prepared. Cells (10^6^ splenocytes or 1–5×10^5^ lymphocytes per well) were co-cultured in the presence of the following antigens: UV-inactivated U112 or U112Δ*iglB* bacterial cells (approximately 1 µg of protein/well), Concanavalin A (ConA, 1 µg/well) as a positive control, and the unrelated antigen hen egg white lysozyme (HEL, 1 µg/well) or medium as negative controls. Mock and U112- vaccinated groups were co-cultured with UV-U112 and the U112Δ*iglB*-vaccinated group was co-cultured with UV-U112Δ*iglB*. Doses for antigenic restimulation were based on previous titrations from selected studies in both mice and rats [12], [27], [48] and were subsequently converted to protein levels using a Bradford assay. After incubation for 72 hr at 37°C, plates were centrifuged and culture supernatants harvested for rat IFN-γ analysis by ELISA (eBioscience, San Diego, CA) per manufacturer's instructions.

### Measurement of Humoral Responses

Eight to nine week old Fischer 344 rats were vaccinated i.t. or orally with either 10^5^ CFU U112, 10^7^ CFU U112Δ*iglB*, or PBS (mock-vaccinated). For serum antibody titers (n = 6 per group), rats were rested for 28 days and bled via the tail vein to obtain sera. Briefly, 96-well plates were coated and incubated overnight with 10^6^ CFU/well UV-inactivated bacteria (U112 for PBS and U112-vaccinated groups, and U112Δ*iglB* for the U112Δ*iglB*-vaccinated group) or with 100 ng/well of the unrelated antigen HEL, all diluted in sodium bicarbonate buffer (pH 9.5). Plates were washed with PBS+0.05% tween-20 using an automated plate washer (BioTek) and blocked with PBS+10% FBS for 2 hr at room temperature. Serum was serially diluted across plates and incubated at room temperature for 2 hr. Plates were washed, incubated for 1 hr with secondary antibody (anti-rat total antibody (including IgA), IgG1, or IgG2a conjugated to horseradish peroxidase, Southern Biotech), washed a second time, and subsequently tetramethylbenzidine substrate (BD Biosciences) was added for color development. Plates were read on an ELISA plate reader (BioTek) at 630 nm. Reciprocal serum dilutions corresponding to 50% maximal binding were used to obtain titers for each animal as in previous studies [27], [48]. These values were then used to obtain means and standard deviation for each experimental group. No binding of immune serum was detected in plates coated with HEL.

For measurement of intestinal antibody responses (n = 6 per group), fresh fecal specimens (normalized to 0.1 g/rat) were collected in protease inhibitor solution (Roche Applied Science, Indianapolis, IN) on day 28. Samples were vortexed vigorously and supernatants collected by centrifugation were assayed for humoral responses (total antibody, IgM or IgA) by ELISA. For collection of bronchioalveolar lavage fluid (BALF), rats (n = 3 per group) were sacrificed and an incision made from the chin to the middle of chest to expose the trachea. A small cut was made in the trachea to allow insertion of an 18-gauge catheter (Exelint, Los Angeles, CA) which was tied in place by a small piece of suture. A 3 mL syringe filled with 1× sterile PBS was attached to the catheter and 1 mL PBS gently injected into the lungs and removed to obtain BALF. The PBS lavage was repeated a total of 3 times, and the BALF centrifuged to remove cells and the supernatant analyzed by ELISA. Due to the small amount of antibody in the BALF and fecal supernatant samples and the large dilution factor in acquiring them (from 3 washings of lungs to collect BALF and from the respective dilution of the fecal pellets in protease inhibitor cocktail), these samples were tested undiluted, and were subsequently reported as OD_630_ values. Means were obtained by taking the average OD_630_ reading of the three animals from each group, and p-values were obtained by two-way ANOVA of OD values of the 3 respective groups. IgM responses in fecal supernants were minimal (data not shown), and no responses were detected in BALF or fecal supernatants to plates coated with HEL.

### Statistics

Statistical analysis was performed using SigmaStat software. The Kaplan-Meier test was used for statistical analysis of survival experiments and student's *t* test for determination of differences in intramacrophage replication and organ burden, as well as antibody and IFN-γ production. Data are represented as mean ± standard deviation from each group.

## Results

### Characterization of F344 Alveolar Macrophages

**Figure 1:**
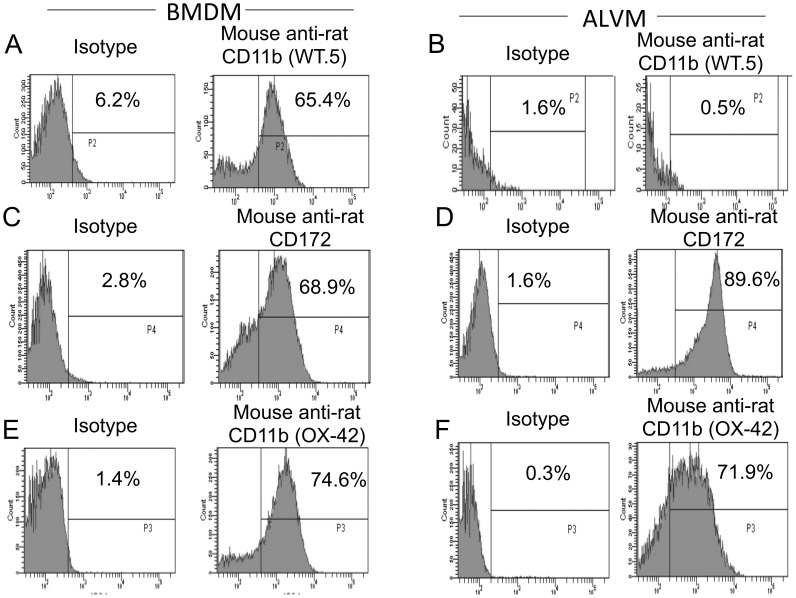
Flow cytometry characterization of bone marrow derived macrophages (BMDM) *vs*. alveolar macrophages (ALVM). Cells were isolated from either bone marrow obtained from Fischer 344 rat femurs (panel A, C, E) or following bronchioalveolar lavage (panel B, D, F) and cultured as described (n = 3 rats for each determination). Upon reaching optimal differentiation, macrophages were stained with Alexa Fluor 647 (AF 647) conjugated mouse anti-rat CD11b (OX-42), fluorescein isothiocyanate (FITC) conjugated mouse anti-rat CD11b (WT.5), or phycoerythrin (PE) conjugated mouse anti-rat CD172 [right column] or respective isotype control [left column] and analyzed by flow cytometry. Results are representative of two independent experiments.

Intramacrophage replication has been used as an important tool to determine attenuation of *F. tularensis* vaccine strains [48]. Therefore, primary alveolar macrophages (ALVM) were utilized to determine whether U112Δ*iglB* was attenuated in the Fischer 344 (F344) rat. Primary ALVM were phenotypically compared with BMDM using flow cytometry [9] for the following macrophage population markers: CD11b (OX-42) (AbD Serotec, Raleigh, NC) [51], CD11b (WT.5) (BD Biosciences, San Diego, CA) [52], [53], and CD172 (also designated as OX41; BD Biosciences) [51]. As shown in Fig. 1A & B, the two types of macrophages could be distinguished by WT.5 (anti-CD11b^+^), with BMDM 65.4% positive while ALVM exhibited 0.5% positive staining as compared to respective isotype controls (6.2 and 1.6%, respectively). In contrast to the differential WT.5 staining, both BMDM and ALVM were positive for CD172 (68.9 and 89.6% respectively) and OX-42 (74.6 and 71.9% respectively) when compared to corresponding isotype controls (2.8 and 1.6%; 1.4 and 0.3%, respectively). Thus, F344 ALVM are a distinct population from that of BMDM as distinguished by CD172^+^ (high) OX-42^+^ (high) and WT.5^−^ (low).

**Figure 2:**
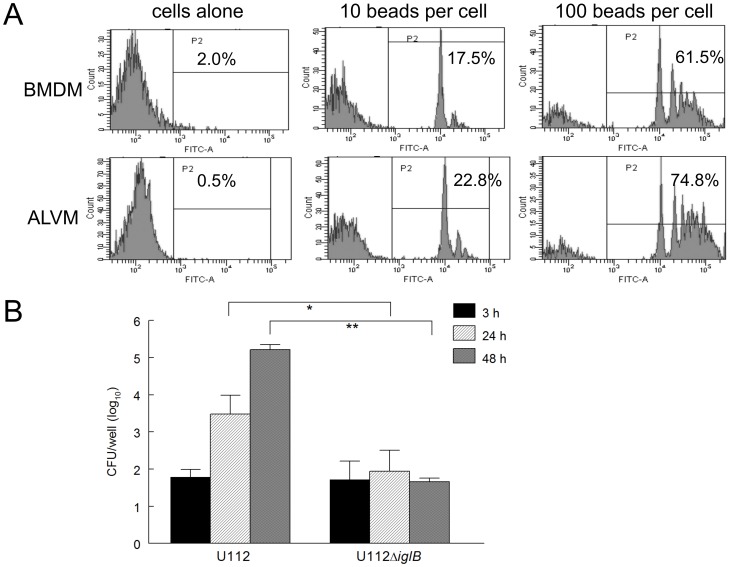
The phagocytic capacity of F344 alveolar macrophages. A) Bone marrow derived macrophages (BMDM) or alveolar macrophages (ALVM) were seeded (1×10^6^ cells/well) into 6-well plates and allowed to adhere. Fluorescent beads were added to each cell type at either 10 or 100 beads/cell and incubated for 2 hr to allow for phagocytosis. Cells were washed to remove unphagocytosed beads and then stained for flow cytometry analysis. B) ALVM (2×10^5^ cells/well) were seeded, allowed to adhere, and infected for 2 hr with 10 MOI of either U112 or the live attenuated defined mutant strain U112Δ*iglB*. Cells were subsequently treated with gentamicin for one hour to kill any remaining extracellular bacteria and incubated at 37°C for 48 hr. At defined time points (3, 24, and 48 hr), cells were lysed with 0.2% (w/v) deoxycholate and dilution plated to enumerate intracellular bacteria. Differences between U112 and U112Δ*iglB* at 24 and 48 hr were significant (*p<0.05 and **p<0.005, respectively). Results are representative of two separate experiments.

In order to assess the functionality and potential differences in phagocytosis capabilities of the two macrophage populations, the phagocytic capacity of ALVM and BMDM was evaluated using a fluorescent bead uptake assay as previously described [12]. Briefly, BMDM and ALVM (1×10^6^ cells/well) were seeded into 6-well plates and allowed to adhere. Fluorescent labeled-beads (FITC conjugated) were added to both cell populations at either 10 or 100 beads/cell and incubated for 2 hr for uptake. Cells were washed to remove unphagocytosed beads, stained and subjected to flow cytometry analysis. These analyses (Fig. 2A) revealed that ALVM exhibited increased uptake of labeled beads compared to BMDM (22.8 *vs* 17.5% at 10 beads/cell and 74.8 *vs.* 61.5% at 100 beads/cell, respectively). There were minimal levels of FITC fluorescence observed in BMDM and ALVM cells cultured without beads (2.0 and 0.5%, respectively). Intramacrophage replication of the WT U112 and mutant U112Δ*iglB* in F344 ALVM cells also was compared. Alveolar macrophages (2×10^5^ cells/well) were infected (10 MOI) for 2 hr with U112 or U112Δ*iglB*, and then pulsed with gentamicin for an additional 1 hr to kill any extracellular bacteria and subsequently incubated for a total of 48 hr. At defined intervals (3, 24, and 48 hr), cells were lysed with 0.2% (w/v) deoxycholate followed by dilution plating. As shown in Fig. 2B, U112 replicated robustly (∼3 logs) over the 48 hr time course. In contrast, minimal increases were observed for U112Δ*iglB* over 48 hr, with significant differences between U112 and U112Δ*iglB* at 24 and 48 hr (*p*<0.05 and *p*<0.005, respectively). These results, which were similar to intramacrophage replication in BMDM (data not shown), extend our previous findings using murine macrophages [48] and further confirm the attenuation of U112Δ*iglB* in F344 ALVMs.

### *In vivo* Growth and Dissemination of Vaccine Strain

**Figure 3:**
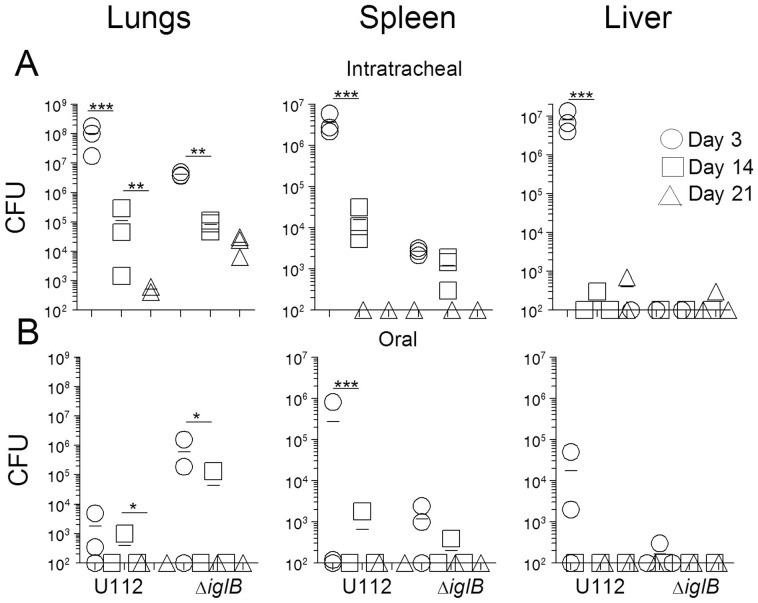
*In vivo* dissemination of U112 and U112Δ*iglB* in Fischer 344 rats. Rats were i.t. (panel A) or orally (panel B) vaccinated with either 10^5^ CFU U112 or 10^7^ CFU U112Δ*iglB*. At defined time points (days 3, 14, and 21 post vaccination), rats (n = 3 per group/time point) were euthanized for collection of lungs, spleens, and livers. Organs were homogenized and dilution plated on TSA plus cysteine to enumerate organ bacterial burdens. Significant differences in burden over the time course are noted (* denotes *p*<0.05, ** denotes *p*<0.01). Results are representative of two separate experiments.

To determine the dissemination profile of U112Δ*iglB* and elucidate appropriate time points for analysis of immune responses from vaccination, bacterial organ burdens were determined following mucosal vaccination. Rats (n = 3 per group/time point) were vaccinated either i.t. or orally with 10^5^ CFU U112 or 10^7^ CFU U112Δ*iglB*. Animals were euthanized at days 3, 14 or 21 for collection of lungs, spleens, and livers, with organs analyzed in all animals regardless of route of vaccine administration. Organs were homogenized and dilution plated to determine bacterial burdens. As shown in Fig. 3A, significant numbers of bacteria were recovered on day 3 from the lungs of rats vaccinated i.t. with either U112 (mean = 1.0×10^8^ CFU) or U112Δ*iglB* (mean = 4.0×10^6^ CFU). Bacterial dissemination occurred in both i.t. vaccinated groups to the spleens (means of 3.6×10^6^ and 2.7×10^3^ CFU for U112 and U112Δ*iglB* groups respectively) and to the liver in the U112-vaccinated group only (8.0×10^5^) by day 3. Bacteria were present but significantly reduced by day 14 in all organs of i.t. U112-vaccinated animals and in lungs of U112Δ*iglB*-vaccinated animals (* denotes *p*<0.05, ** denotes *p*<0.01, *** denotes *p*<0.001). Spleens and livers were cleared by day 21 with lower burdens detected in the lungs of intratracheally-vaccinated animals (limit of detection of assay = 100 CFU). In contrast, orally vaccinated groups (Fig. 3B) each had only 2 out of 3 animals with any bacterial burden on day 3. Slight dissemination from intestines to lungs (means of 1.7×10^3^ (U112) and 5.9×10^5^ CFU (U112Δ*iglB*)), spleen (2.7×10^5^ or 1.1×10^3^ for U112 and U112Δ*iglB* respectively), and liver (1.7×10^4^ and 100 CFU for U112 and U112Δ*iglB* respectively) occurred by day 3. Dissemination to lungs in the orally vaccinated animals may actually have occurred from aspiration of part of the inocula and not by direct dissemination from the intestines. By day 14, negligible amounts of bacteria were recovered from all organs in the orally vaccinated groups, with a significant decrease in burden in the lungs of bothgroups (*p*<0.05) and in the spleens of U112-vaccinated animals (*p*<0.001). Bacteria were completely cleared from all organs by day 21. Regardless of route or vaccine strain, burdens were lower on days 14 and 21 as the strains are cleared from the animal.

### Antigen Specific Cellular Responses Following Mucosal Vaccination

In order to compare the efficacy of two different mucosal routes of immunization with U112Δ*iglB*, F344 rats (n = 3 per group) were vaccinated i.t. or orally with either 10^5^ CFU U112, 10^7^ CFU U112Δ*iglB* or mock vaccinated with PBS and rested for 14 or 28 days. Cellular responses were measured at two time points because at the earlier 14 day time point spleens still showed presence of bacteria in burden assays. As spleens were cleared by day 21 (Figure 3), cellular responses also were measured at day 28 to ensure no bacteria was present in organs at this time point. Rats were euthanized as described previously for collection of spleens and draining lymph nodes. Single-cell suspensions were prepared and cultured for 72 hr in the presence of approximately 1 µg of protein from either UV-inactivated U112 (for mock and U112 vaccinated groups) or UV-inactivated U112Δ*iglB* (for U112Δ*iglB* vaccinated groups). Supernatants were analyzed by ELISA for the production of IFN-γ.

As shown in Table 1, splenocytes from rats vaccinated i.t. or orally with U112 produced significant (*p*<0.001) levels of antigen-specific IFN-γ (16,258±1355 and 29,840±1158 pg/mL, respectively) compared with mock-vaccinated animals at 14 days and are able to maintain IFN-γ production upon recall at 28 days (1050±28 and 146±28 pg/mL for i.t. and orally vaccinated animals, respectively). Splenocytes from rats vaccinated with U112Δ*iglB* also produced significant amounts of IFN-γ (2234±329 pg/mL i.t. and 151±83 pg/mL orally, *p*<0.01 compared to mock at day 14, albeit at lower levels than U112. Only U112Δ*iglB* intratracheally-vaccinated animals produced IFN-γ at day 28 (53±10 pg/mL). Rats vaccinated with U112 also produced significant (*p*<0.001 for both routes versus mock groups) amounts of IFN-γ (1103±99 pg/mL i.t. and 2967±7 pg/mL orally) within cervical lymph nodes (CLN) at day 14, whereas rats receiving U112Δ*iglB* produced minimal amounts of IFN-γ at this time point by either route of vaccine administration. A similar pattern was seen at day 14 in mesenteric lymph nodes (MLN,), with U112 i.t. vaccination generating 3715±207 pg/mL of IFN-γ (*p*<0.001 compared to mock) and orally resulting in 2765±31 pg/mL compared to low levels of IFN-γ produced from U112Δ*iglB*-primed MLN. Due to limitations in the amount of cells recovered from CLN and MLN at day 28 (1×10^5^ cells/well at this time point compared to 5×10^5^ cells/well at day 14) we cannot conclude that IFN-γ is not produced in the draining lymph nodes upon restimulation at day 28, however, under these conditions we did not see any IFN-γ production in our draining lymph node ELISAs at this time point. Across the board, there was negligible IFN-γ production in any cells (regardless of route or vaccine) cultured with media or with the unrelated antigen hen egg lysozyme (HEL. data not shown) and potent responses (*p*<0.001) comparable across all groups when tissues were cultured with the mitogen Concanavalin A.

**Table 1:** Antigen-specific cellular responses following mucosal vaccination.

IFN-γ Production		DAY 14						DAY 28					
Post													
Immunization		PBS		U112		U112Δ*iglB*		PBS		U112		U112Δ*iglB*	
(pg/mL)		Mean	SD	Mean	SD	Mean	SD	Mean	SD	Mean	SD	Mean	SD
**I.T.**	**Spleen**	<30	N/A	16258	1355	2234	329	<30	N/A	1051	28	54	10
	**CLN**	<30	N/A	1103	99	<30	N/A	<30	N/A	<30	N/A	<30	N/A
	**MLN**	540	526	3716	207	101	52	<30	N/A	<30	N/A	<30	N/A
**Oral**	**Spleen**	<30	N/A	29840	1158	151	83	<30	N/A	146	28	<30	N/A
	**CLN**	<30	N/A	2967	7	<30	N/A	<30	N/A	<30	N/A	<30	N/A
	**MLN**	<30	N/A	2766	31	32	N/A	<30	N/A	<30	N/A	<30	N/A

Rats were vaccinated either i.t. (top panel) or orally (bottom panel) with either 10^5^ CFU U112, 10^7^ CFU U112Δ*iglB*, or mock-vaccinated with PBS, and rested for 14 or 28 days. Rats were euthanized and spleens (10^6^ cells/well) and draining lymph nodes (CLN and MLN) collected (day 14: 5×10^5^ cells/well and day 28: 1×10^5^ cells/well). Single cells were cultured for 72 hr in the presence of approximately 1 µg of protein from either UV-inactivated U112 (for U112 and mock vaccinated groups), or UV-inactivated U112Δ*iglB* (for U112Δ*iglB* vaccinated group). Cells were then separated by centrifugation and supernatants were collected and assayed for IFN-γ production by ELISA (shown here in pg/mL, limit of detection is 30 pg/mL). Results are representative of two independent experiments at each time point. CLN = cervical lymph nodes, MLN = mesenteric lymph nodes, SD = standard deviation, NA = not applicable.

### Humoral Responses Following Mucosal Vaccination

**Figure 4:**
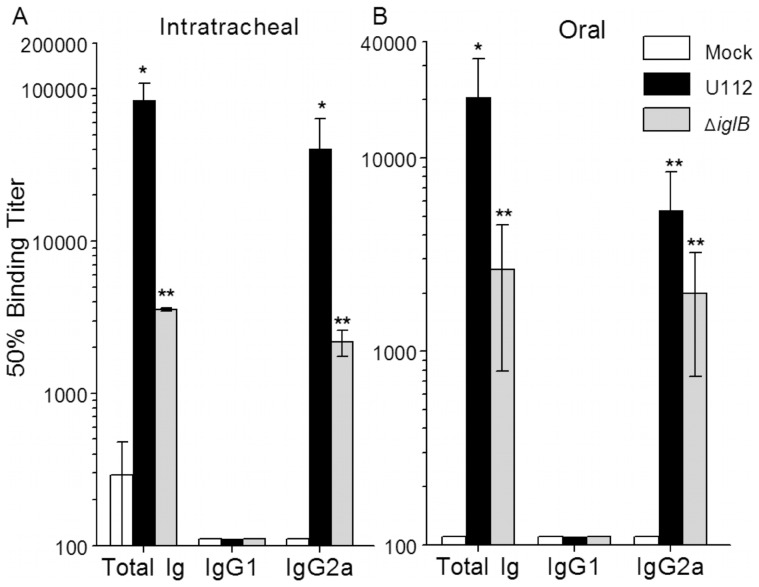
Serum antibody responses following mucosal vaccination. Animals (n = 6 per group) were vaccinated i.t. (panel A) or orally (panel B) with either 10^5^ CFU U112, 10^7^ CFU U112Δ*iglB*, or mock vaccinated with PBS and rested for 28 days. Serum was analyzed by ELISA to obtain 50% binding titers. Titers from both vaccinated groups, regardless of route, were shown to be significant compared to mock (**p<0.001, *p<0.01). Results are representative of two independent experiments.

**Figure 5:**
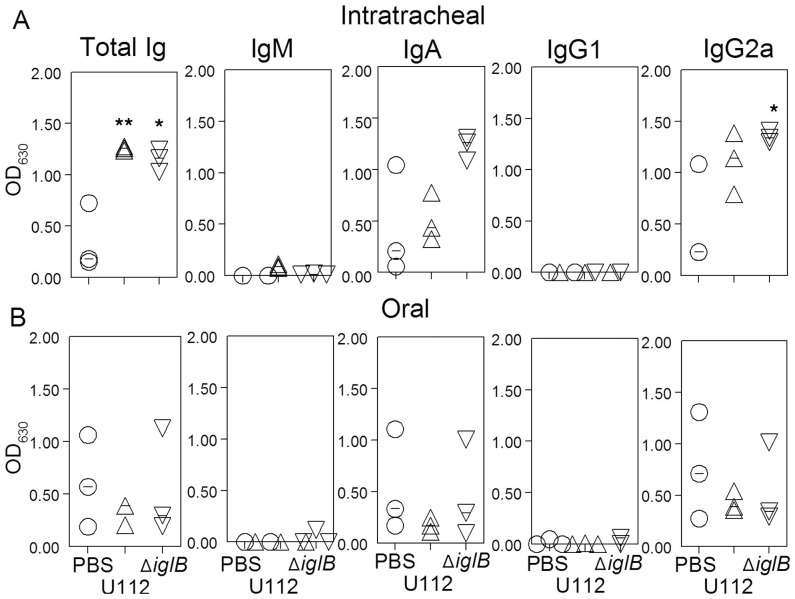
Respiratory antibody response following mucosal vaccination. Animals (n = 3 per group) were vaccinated i.t. (top panel) or orally (bottom panel) with either 10^5^ CFU U112, 10^7^ CFU U112Δ*iglB*, or mock vaccinated with PBS and rested for 28 days. Animals were euthanized to obtain bronchioalveolar lavage fluid (BALF) which was assayed by ELISA. Significant differences were observed between U112 and U112Δ*iglB* i.t. vaccinated and respective mock groups for total Ig and IgG2a (*p<0.05, **p<0.01). Animals primed with U112Δ*iglB* exhibited responses which were comparable to or higher than that observed for U112-primed rats. Horizontal lines represent mean of each group. Results are representative of two separate experiments.

**Figure 6:**
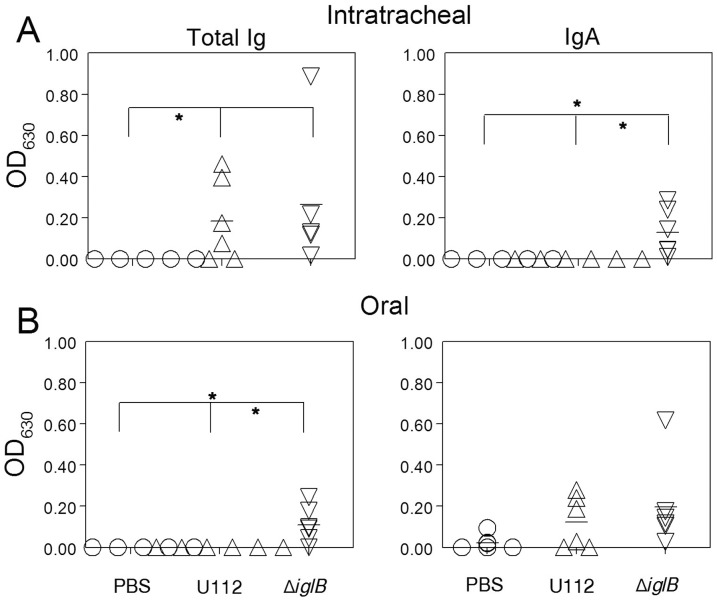
Intestinal antibody response following mucosal vaccination. Animals (n = 6 per group) were vaccinated i.t. (A, top panel) or orally (B, bottom panel) with either 10^5^ CFU U112, 10^7^ CFU U112Δ*iglB*, or mock vaccinated with PBS and rested for 28 days. Fecal specimens (0.1 g/rat) were collected, processed and supernatants analyzed by ELISA for total immunoglobulin response and IgA. Vaccinated animals produced higher levels of IgA and total Ig (*p<0.05) compared to the mock-vaccinated group. Animals primed with U112Δ*iglB* exhibited responses which were comparable to or higher than that of U112-vaccinated rats. Horizontal lines represent mean of each group. Results are representative of two independent experiments.

Humoral responses within the systemic and mucosal compartments were analyzed at 28 days following vaccination. Rats (n = 6 per group) were vaccinated i.t. or orally with either 10^5^ CFU U112, 10^7^ CFU U112Δ*iglB*, or mock vaccinated with PBS. Prior to bacterial challenge, animals were bled (day 28 post-vaccination) and serum analyzed by ELISA. F344 rats vaccinated i.t. with U112 exhibited significant (*p*<0.01) induction of total Ig and IgG2a with minimal production of IgG1 in contrast to mock-vaccinated animals (Fig. 4A). A similar profile, albeit at lower levels, was observed in rats vaccinated i.t. with U112Δ*iglB* (*p*<0.001 compared to mock-vaccinated group). A comparable but somewhat reduced antibody profile was observed following oral vaccination with the WT and mutant strains of bacteria (Fig. 4B). In all cases, there was minimal reactivity to the unrelated HEL antigen (data not shown) and negligible antibody production in mock-vaccinated animals (detection limit ∼100). Antibody responses on day 28 at the sites of vaccination also were evaluated by collection of bronchioalveolar lavage fluid (BALF) (Fig. 5) and fecal supernatants (Fig. 6) from vaccinated rats. As shown on Fig. 5A, rats vaccinated i.t. with U112 exhibited increased mean levels of total antibody, IgA and IgG2a in contrast to mock-vaccinated animals (*p<0.05, **p<0.01). Rats vaccinated i.t. with U112Δ*iglB* showed comparable levels of total antibody and IgG2a, and increased levels of IgA when compared with animals vaccinated with U112. In contrast, oral vaccination gave rise to lower levels of total antibody, IgA and IgG2a compared to rats primed i.t. (Fig. 5B). There was a greater induction of fecal IgA response following i.t. vaccination with U112Δ*iglB* than with U112 (Fig. 6A). In contrast, there were comparable levels of fecal IgA production following oral vaccination with U112 or U112Δ*iglB* (Fig. 6B), with minimal IgM production across all groups (data not shown). As expected, a minimal antibody response was observed in mock (PBS)-vaccinated rats.

### Pulmonary Protection Conferred by Mucosal Vaccination

**Figure 7:**
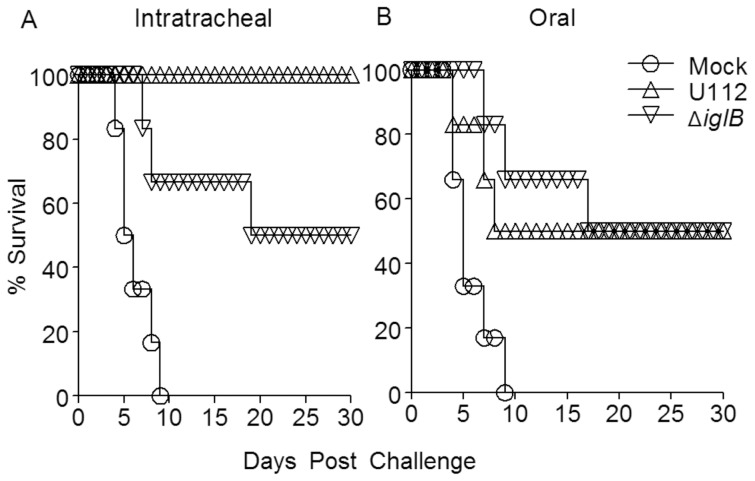
Protective immunity conferred by mucosal vaccination. Animals (n = 6 per group) were vaccinated either i.t. (panel A) or orally (panel B) with either 10^5^ CFU U112, 10^7^ CFU U112Δ*iglB*, or mock-vaccinated with PBS and rested for 30 days. Rats were challenged i.t. with 1.25×10^4^ CFU SCHU S4 (approx. 25 LD_50_) and monitored daily for morbidity and mortality. Kaplan-Meier survival analysis revealed protection following immunization with U112 by both i.t. and oral routes were significant (*p*<0.001 and *p*<0.005, respectively) over mock immunization. Protection conferred by U112Δ*iglB* vaccination was significant over mock control (*p*<0.01 (i.t.) and *p*<0.005 (oral)). Results are representative of two independent experiments.

The efficacy of both routes of vaccination in providing protection against lethal pulmonary challenge with the highly human virulent SCHU S4 strain of *F. tularensis* was compared (Fig. 7). Rats (n = 6 per group) were vaccinated i.t. or orally with either 10^5^ CFU U112, 10^7^ CFU U112Δ*iglB*, or mock-vaccinated with PBS and rested for 30 days prior to i.t. challenge with 1.25×10^4^ CFU (approximately 25 LD_50_) of SCHU S4. As shown in Fig. 7A., i.t. vaccination with the WT strain U112 provided complete protection (100% survival, *p*<0.001 compared to mock-vaccinated animals) against the lethal SCHU S4 pulmonary challenge in agreement with previously published results [12], whereas animals vaccinated orally with U112 exhibited 50% survival (*p*<0.05 compared to mock, Fig. 7B). In contrast, regardless of route, vaccination with U112Δ*iglB* conferred 50% protection against subsequent pulmonary challenge (*p*<0.01 for i.t. group and *p*<0.005 for oral group compared to mock, Fig. 7). Mock-vaccinated rats succumbed to the bacterial challenge by day 10. These results, which are consistent between replicate experiments, clearly indicate the feasibility of developing a defined U112 vaccine strain and the efficacy in providing protective immunity against pulmonary tularemia.

## Discussion

Mucosal immunization has been exploited as a successful route for vaccination against a variety of pathogens that infect the respiratory and gastrointestinal tracts [21], [22], [26], [29]. The licensed intranasal vaccine FluMist has been used successfully against seasonal influenza; and oral vaccination platforms also have been effectively used against other pathogens, most notably poliovirus (Sabin vaccine), typhoid fever (Ty21a vaccine), and rotavirus [55]. In this study, we sought to analyze and compare two routes (i.t. and oral) of mucosal vaccination in the Fischer 344 rat utilizing a live attenuated vaccine strain (U112Δ*iglB*) that has been previously characterized in the mouse model of *F. tularensis* [48]. This live attenuated strain lacks the *iglB* gene within the iglABCD operon of the *Francisella* pathogenicity island (FPI), consisting of 17 genes. Two copies of the FPI are found in the highly human virulent subsp. *tularensis* and *holarctica*, whereas a single copy of the FPI is found in subsp. *novicida* [45]. There is >97% homology between the FPIs across subspecies, and genes of the FPI are required for intramacrophage replication, phagosomal escape, and virulence [45], [46], [56]–[61]. The *iglB* gene has been shown to have homology in other bacteria such as *Vibrio cholerae*, *Salmonella enterica*, and *Rhizobium leguminosarum* [62] and been demonstrated to be part of a type VI secretion system in other species [63], [64] as well as in *F. tularensis* [46], [47].

This study utilized intratracheal vaccination in the rat, which would be an impractical route of vaccination for humans, when compared to the more common intranasal route. The complex physiology of the rat respiratory system suggests that intratracheal vaccination may be a more effective mechanism to ensure the vaccination and challenge inocula reach the lungs in this animal model. In contrast, intranasal vaccination of the rat would lead to increased deposition of inocula in the nasal passages due to the complex turbinate structure which is characteristic of rodents and is absent in humans [54]. Thus, although intratracheal vaccination may be impractical for use in humans, this route may be the most effective one in the rat to compare to intranasal routes in humans.

In this study, the overall induction of antigen–specific cellular and humoral responses was lower in U112Δ*iglB*-immunized rats than those receiving the parental strain U112. This difference is most likely related to the level of attenuation observed for alveolar intramacrophage replication and subsequent priming of the immune system. Likewise, cellular and humoral responses were observed to be higher for U112 at the priming sites for each respective route (cervical lymph nodes [day 14] and BALF for i.t. vaccination; mesenteric lymph nodes [day 14] and intestines for oral vaccination). Additionally, antigen-specific IFN-γ was produced at distal sites at day 14, illustrating the commonality of the mucosal immune system. Our analyses revealed that both U112 and U112Δ*iglB* vaccinated rats exhibited a Th1-driven, systemic humoral response (IgG2a over IgG1) which differed significantly from the mouse, where mixed serum responses of both IgG1 and IgG2a isotypes is exhibited [48]. A similar polarized antibody response (high levels of total Ig, IgA, and IgG2a and minimal IgM or IgG1) was observed at the sites of priming (lung or intestinal compartment depending on vaccination route). Rats also exhibited a lower, but intact antibody response at distal mucosal sites which mirrored the cellular IFN-γ responses.

Importantly, immunization with U112Δ*iglB*, regardless of route, was able to provide 50% protection against subsequent pulmonary challenge with 25 LD_50_ (1.25×10^4^ CFU) of the highly human virulent *F. tularensis* strain SCHU S4. Moreover, when U112Δ*iglB* was administered orally, the observed levels of protective immunity was equal to that conferred by WT U112 providing further evidence that this defined mutant strain may serve as a promising candidate for further investigation. Interestingly, our cellular responses at day 28 correlate with the survival, as the U112 i.t. group (which had 100% survival following SCHU S4 challenge) produced significantly higher amounts of IFN-γ compared to the other three vaccine treatments/routes with which comparable IFN-γ production and resulting 50% survival.

Differences in survival between the two mucosal routes following SCHU S4 challenge may be due to a variety of factors. Oral vaccination, as opposed to immunization by the intratracheal route, may involve compounding factors which could be responsible for equalizing the immune responses generated from a lower-dose vaccination with the WT U112 and higher dose vaccination with the attenuated mutant U112Δ*iglB* strain. For example,organisms may not survive the highly acidic pH of the stomach, or they may be lost from the digestive tract as a consequence of peristalsis and fluid flow clearing mechanisms. In contrast, intratracheal administration places organisms directly onto the mucosal surfaces of the rat lung and thus more bacteria may be retained following i.t. immunization when compared to the GI tract.

Given that LVS has been examined extensively [13], [27], [65]–[69] as the prototypic vaccine candidate, we also evaluated the efficacy of oral LVS vaccination in this model. LVS has previously been documented to provide protection by parenteral (intradermal and subcutaneous) and mucosal (intratracheal) routes in the F344 rat [13] and has a similar LD_50_ to U112Δ*iglB* within the rat model (LD_50_ of both strains >10^7^ CFU by the pulmonary route). We found that oral LVS vaccination conferred complete protection against pulmonary SCHU S4 (approximately 25 LD_50_) challenge (Arulanandam and Signarovitz, unpublished observations).

Despite the high level of protection conferred by WT U112, this bacterium would most likely not be a successful candidate for vaccination against tularemia due to its wild-type nature and the obvious morbidity observed following vaccination of rats. Specifically, F344 rats vaccinated i.t. with 10^5^ CFU U112 in this study were visibly stressed and ill for 7–10 days following immunization, with symptoms including ∼10% weight loss, ruffled fur, hunched posture, and periorbital porphyrin production. Such severe morbidity in immunocompetent hosts would likely prevent administration of U112 to immunocompromised individuals. In contrast, vaccination with a hundred-fold higher dose of U112Δ*iglB* caused no adverse effects or visible morbidity to rats, and yet this mutant was still able to induce antigen-specific cellular and humoral responses which generated protection against subsequent SCHU S4 challenge. It is likely that booster doses of this mutant strain would increase the degree of protective efficacy. These results collectively suggest the feasibility of developing targeted oral-based attenuated mutant vaccine strains for immunization against *F. tularensis* and provide impetus for further refinement of *novicida*-based vaccines, given the ease of its genetic manipulation.

To this end, U112Δ*iglB* is the only *F. novicida*-based live attenuated vaccine strain that has been shown to provide heterologous protection against pulmonary LVS and SCHU S4 challenge in the mouse model [33]. The majority of *F. novicida*-based putative vaccines, including other FPI mutants such as Δ*iglC* [5], Δ*pdpB* [70], and FPI regulator Δ*mglA* [71], have only been tested against homologous U112 challenge. The two *F. novicida*-based vaccines tested against SCHU S4 were non-FPI mutants, namely Δ*purF* [8] and Δ*pmrA* [72], which exhibited no protective efficacy following pulmonary challenge, and no SCHU S4-based FPI mutant has provided protection against subsequent Type A challenge. For example, SCHU S4Δ*iglC*, when administered at high doses by either intradermal or oral routes, afforded no protection against subsequent pulmonary SCHU S4 challenge [6], [34]. SCHU S4Δ*iglB* and SCHU S4Δ*iglD* also demonstrated marginal protection against subsequent pulmonary challenge [73]. Levels of protection afforded by non-FPI LVS [2], [35], [74] or SCHU S4-based [6], [34], [75] mutants varied within the limited challenge dose of less than 10 to 100 CFU, illustrating the high sensitivity of the mouse in contrast to the rat, and consequent limitation of this model for vaccine efficacy studies. The success of mucosal vaccination in the F344 rat as demonstrated here and by others [12], [13] may involve microfold cells (M-cells). These cells are predominantly found in the follicle-associated epithelium (FAE) of intestinal Peyer's patches (PP), which are components of the larger intestinal GALT (gut-associated lymphoid tissue) [76], but also can be found in isolated lymphoid follicles, the appendix, and in MALT sites outside the gastrointestinal tract including the nasal passages. Furthermore, M-cells have distinctive morphological features such as a poorly organized brush border, irregular microvilli, and a thin glycocalyx suggesting that they do not play a role in intestinal digestion or absorption [76]. Importantly, M-cells can serve as antigen sampling sites and contain a distinct basal invagination in which live and non-replicating pathogens are presented to lymphocytes, dendritic cells, and macrophages [77]. Our ongoing studies include enhancing the M-cell tropism of defined *F. tularensis* vaccine strains, such as U112Δ*iglB*, to further increase vaccine efficacy and optimal protective immunity.
